# Association between homocysteine levels and calcific aortic valve disease: a systematic review and meta-analysis

**DOI:** 10.18632/oncotarget.23938

**Published:** 2018-01-03

**Authors:** Guandi Wu, Jiayi Xian, Xi Yang, Jiaying Li, Jichen Liu, Wenhui Dong, Shuwen Su, Jun Li, Yan Tu, Jian Peng, Dingli Xu, Qingchun Zeng

**Affiliations:** ^1^ Department of Cardiology, First Clinical Medical College, Nanfang Hospital, Southern Medical University, Guangzhou, China; ^2^ Key Laboratory For Organ Failure Research, Ministry of Education of the People's Republic of China, Guangzhou, China

**Keywords:** homocysteine, homocys, calcific aortic valve disease, aortic valve stenosis, meta-analysis

## Abstract

Previous studies have reported inconsistent results regarding the association between homocysteine (Hcy) levels and calcific aortic valve disease (CAVD). We investigate the association between Hcy levels in patients with CAVD and controls by conducting a systematic review and meta-analysis. We conducted a systematic search of studies published prior to the end of March 2017 in the PubMed, Embase, Web of Science, Cochrane Central Register of Controlled Trials and the Chinese Biomedical Literature databases. Eligible studies evaluating plasma Hcy levels in CAVD patients and controls were identified by two independent investigators. Standardized mean difference (SMD) and the corresponding 95% confidence intervals (95% CIs) were estimated using the random-effects model. Ten studies involving 6349 participants were included. Pooled analysis demonstrated that Hcy levels were significantly elevated in patients with CAVD compared with controls (pooled SMD: 0.57, 95% CI: 0.36–0.79). This elevation was more obvious in American and Asian populations than in Turkish populations. Furthermore, Hcy levels were significantly elevated in patients with mild-to-moderate CAVD and severe CAVD. Our results demonstrate that CAVD is associated with elevated Hcy levels.

## INTRODUCTION

Calcific aortic valve disease (CAVD) is the most common valvular disorder among the aging population—roughly one-third of all individuals over an age of 65 have mild CAVD, manifested as aortic valve sclerosis (AVSc) [1]. With the progression of the pathological condition, aortic valve stenosis (AVS) may appear, in which left ventricular outflow obstruction is presented. This end-stage of CAVD leads to life-threatening diseases such as heart failure. It is reported that 2% and 4% of individuals aged over 65 and 85, respectively, develop AVS [2]. Surgical aortic valve replacement or transcatheter aortic valve replacement is required to avoid the risk of death. Currently, no medical therapy has been confirmed to be effective at reversing this process. Therefore, it becomes urgent to understand modifiable risk factors of CAVD to guide prevention and treatment strategies.

Homocysteine (Hcy), a sulfur-containing amino acid, is a branch-point intermediate of methionine metabolism. Methionine from dietary protein is activated by ATP to form S-adenosylmethionine (SAM), the universal methyl-group donor. A subproduct of these methylation reactions is S-adenosylhomocysteine (SAH), which is converted to Hcy by SAH hydrolase [3]. Homocysteine can be further metabolized via two alternative pathways. First, Hcy can be remethylated to form metionine by methionine synthase (MS), which uses methylenetetrahydrofolate reductase (MTHFR) as a methyl donor. In this reaction, vitamins B_12_ and folate are co-factors [4]. Second, in the transsulphuration pathway, Hcy reacts with serine to form cystathionine, catalyzed by the vitamin-B_6_-dependent enzyme, cystathionine β-synthase (CBS). The pathway continues with the synthesis of cysteine [4].

Homocysteine is considered to be an independent risk factor for cardiovascular disease [5]. Previous studies have shown that Hcy is associated with atherosclerosis [6]. Calcific aortic valve disease, which is considered to share similar biological processes with atherosclerosis, involves endothelial dysfunction, lipid infiltration, inflammation, oxidative stress and mineralization of aortic valves [7]. Previous studies have demonstrated an association of abnormal metabolism of calcium and lipid with the presence of CAVD [7, 8]. Because increased Hcy levels can cause endothelial injury [9], Hcy has been hypothesized to initiate the inflammatory process and facilitate a series of reactions in CAVDs.

In recent years, researchers have attempted to test and verify this hypothesis. A number of case-control studies have investigated Hcy levels in patients suffering from CAVD. However, the results have been inconsistent, like the consequence of small sample sizes and variable study populations. A comprehensive analysis of the association of Hcy levels with CAVD has been lacking in the literature. Now, we have carried out a systematic review to provide a more reliable estimate of the relation between plasma Hcy levels and CAVD. Because no cohort studies were available, this meta-analysis assessed an association but did not demonstrate causation between Hcy levels and CAVD.

## RESULTS

### Literature selection

**Figure 1:**
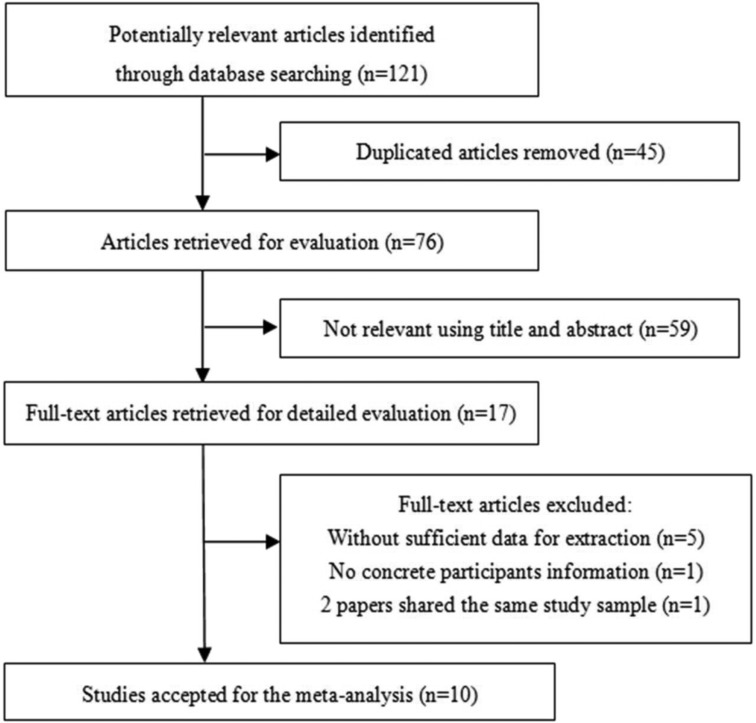
Flow chart of the literature selection process for the meta-analysis

We found 121 potentially relevant articles. After removing duplicate articles and eliminating irrelevant articles by screening their titles and abstracts, 17 articles were passed to the second-stage selection. Two papers shared the same study sample, so the paper with less data [10] was excluded. Ultimately, 10 studies [11–20] satisfied the inclusion criteria and were included in the systematic review. The detailed steps of the literature search are presented in Figure 1. The baseline characteristics and quality assessment of the included studies are listed in Table 1.

**Table 1:** Baseline characteristics and quality assessment of included studies

Study, year	Country	No. patients (M/F)		Age, years		Hcy levels,μmol/L(Mean ± SD)		NOS score
		Cases	Controls	Cases	Controls	Cases	Controls	
Yoram A, 2001[7]	America	140 (85/55)	241 (113/128)	76 (67–85)	63 (57–69)	11.1 (9.1-13.5)	8.9 (7.4–11.1)	8
Novaro GM, 2004[8]	America	AVSc:32 (21/11)	27 (19/8)	AVSc:68 (60–74)	57 (49–70)	AVSc:11.3 (9.3–12.8)	9.2 (8.2–14.6)	7
		AVS:17 (9/8)		AVS:78 (71–83)		AVS:16.6 (12.7–17.8)		
Gunduz H, 2005[9]	Turkey	58 (35/23)	47 (27/20)	64 ± 11	62 ± 13	10.8 ± 3.3	8.1 ± 4.7	7
Bozbas H, 2007[10]	Turkey	112 (75/37)	173 (117/56)	73.0 ± 7.4	68.5 ± 6.7	12.9 (11.1–16.8)	12.3 (10.4-15.4)	7
Ferrari G, 2010[11]	America	33 (15/18)	11 (8/3)	75.9 ± 7.2	55.4 ± 24.2	20.34 ± 2.14	19.23 ± 4.19	6
Sun, 2012[12]	China	101 (36/65)	87 (44/43)	67.0 ± 9.0	59.4 ± 6.9	17.6 ± 8.8	14.9 ± 6.6	7
Yan, 2013[13]	China	116 (44/72)	84 (46/38)	78.2 ± 8.1	68.9 ± 6.8	17.5 ± 8.7	14.7 ± 6.5	6
Guerraty MA, 2015[14]	America	AVSc:515 (253/262)	1023 (557/466)	AVSc:62.3 ± 7.9	53.2 ± 11.7	AVSc:14. 17 ± 4.86	13.54 ± 5.03	7
		AVS:426 (236/190)		AVS:66.5 ± 7.0		AVS:16. 55 ± 7.06		
Zhu, 2015[6, 15]	China	1374 (816/558)	1520 (984/536)	70.9 ± 9.5	54.2 ± 8.0	17.08 ± 9.74	11.65 ± 3.74	7
Liu, 2015[16]	China	106 (70/36)	106 (68/38)	61.9 ± 5.8	60.6 ± 6.2	19.85 ± 7.15	11.97 ± 2.49	7

Abbreviations: M: male; F: female; Hcy: homocysteine; SD: standard deviation; NOS: Newcastle-Ottawa Quality Assessment Scale.

### Association between Hcy levels and CAVD

**Figure 2:**
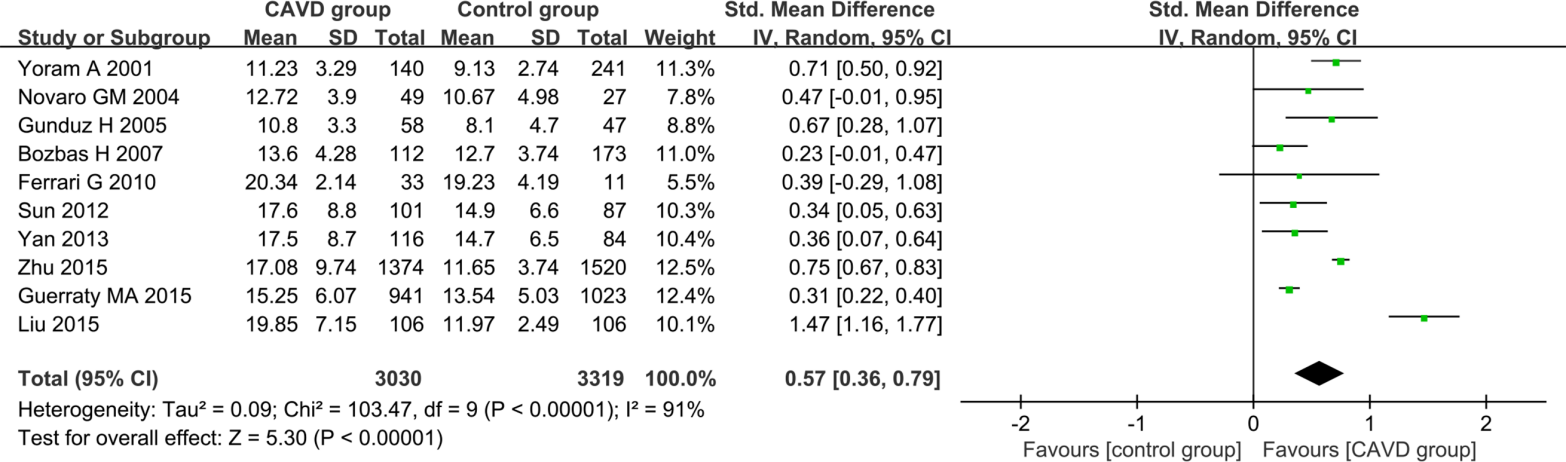
Forest plot of the differences in Hcy levels between CAVD patients and controls Abbreviations: 95% CI, 95% confidence interval.

A total of 3030 CAVD patients and 3319 control subjects were included in our pooled analysis. Overall, CAVD patients had higher plasma Hcy levels than the control participants [pooled standardized mean difference (SMD): 0.57, 95% confidence interval (CI): 0.36–0.79] (Figure 2). We used the random effect model because heterogeneity was considered significant with an I^2^ of 91%.

**Figure 3:**
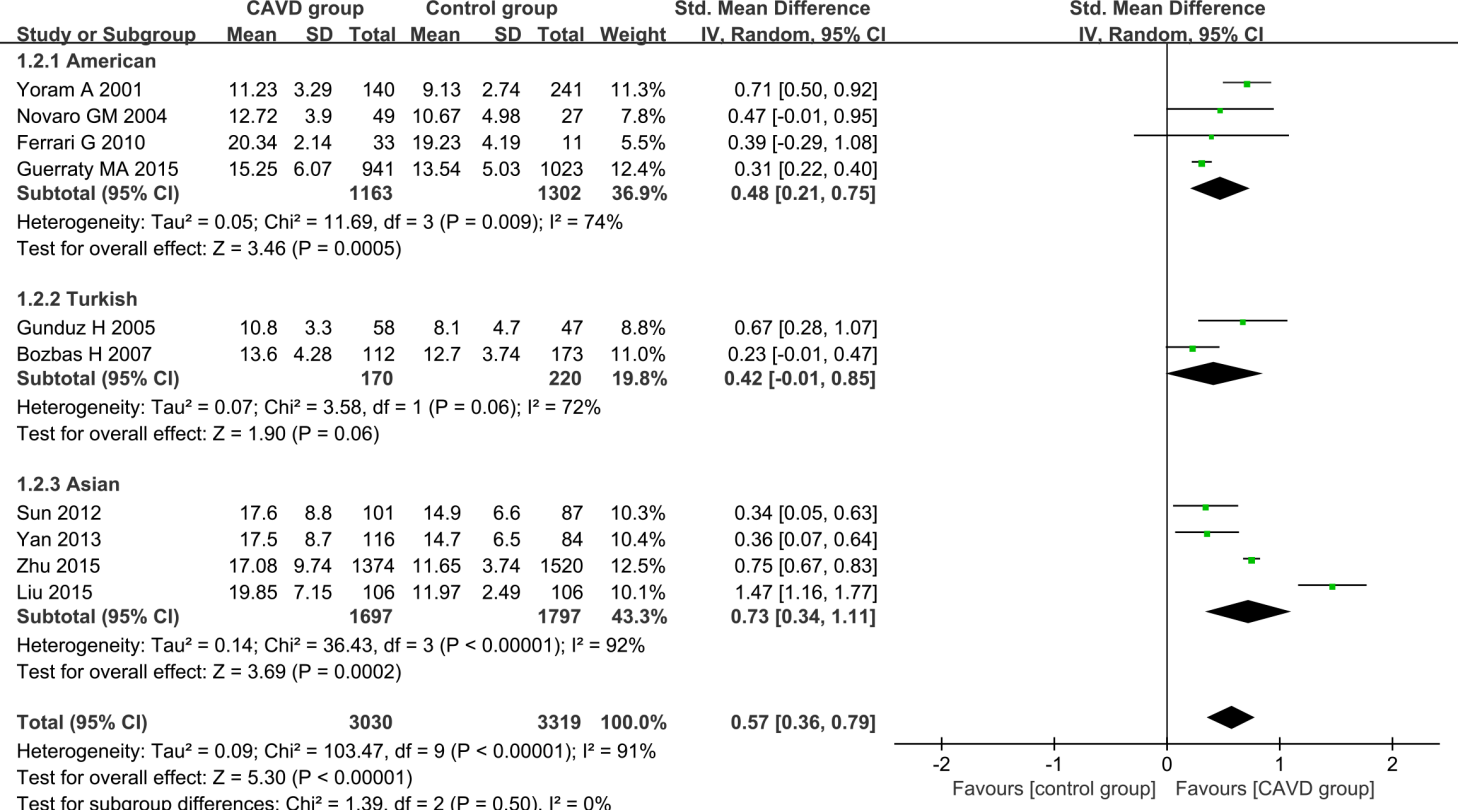
Subgroup analyses for the differences in Hcy levels between CAVD patients and controls of different ethnicities Abbreviations: 95% CI, 95% confidence interval.

**Figure 4:**
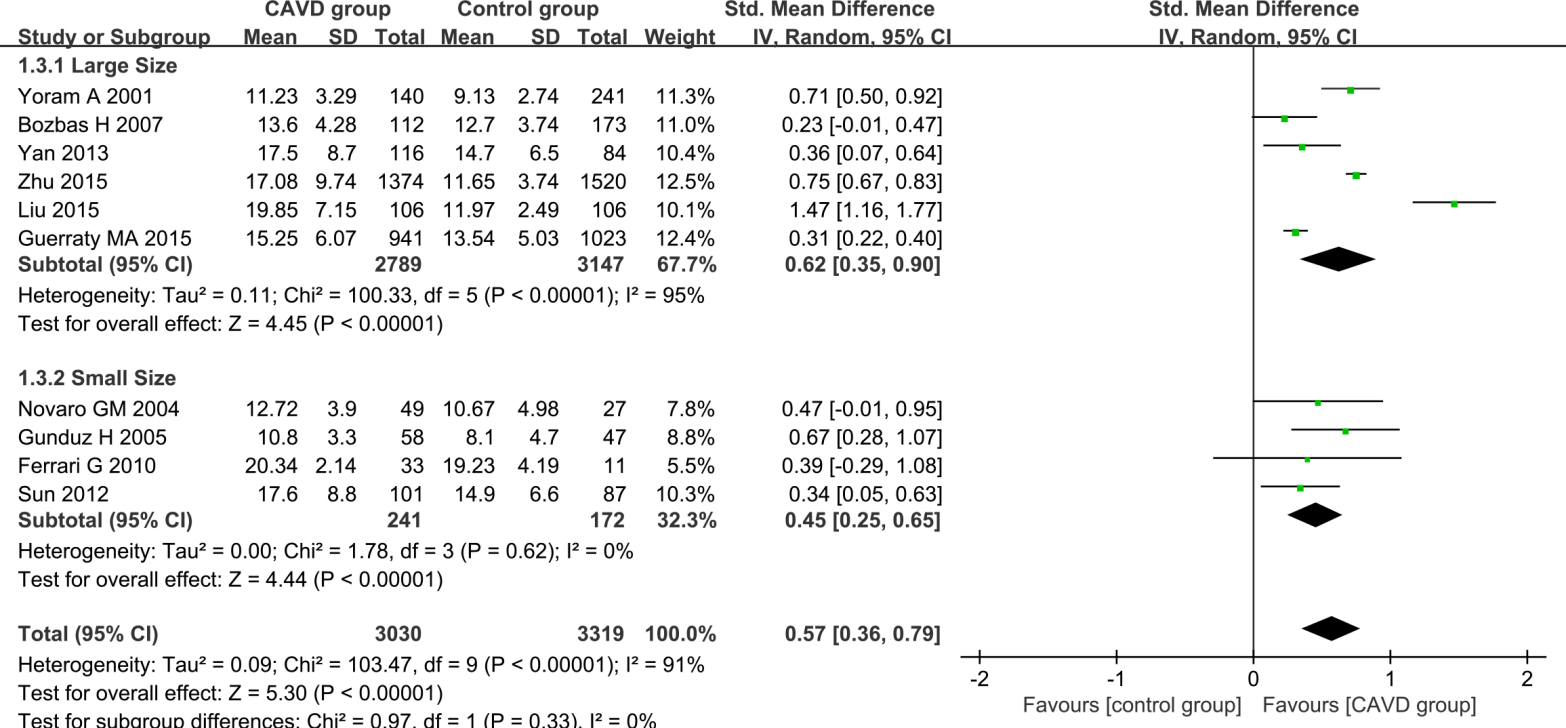
Subgroup analyses for the differences in Hcy levels between CAVD patients and controls in studies with different sample sizes Studies with participant number exceeding 200 were considered as large size studies, and studies with participant number fewer than 200 were considered as small size studies. Abbreviations: 95% CI, 95% confidence interval.

To thoroughly understand the relation between characteristics of CAVD patients and Hcy levels and explore the potential sources of heterogeneity, further subgroup analyses were carried out. Our analyses were based on geographic site (the United States of America, Asia or Turkey) and sample size (≥ 200 or < 200). Random effect models were used in all of the subgroup analyses. In the subgroup analysis of the geographic site, both the American group (SMD 0.48, 95% CI 0.21–0.75) and the Asian group (SMD 0.73, 95% CI 0.34–1.11) exhibited higher plasma Hcy levels among CAVD patients than the controls (Figure 3). The plasma Hcy trended towards a higher level in Turkish CAVD patients than in the controls (SMD 0.42, 95% CI -0.01–0.85) (Figure 3). Different sample sizes were also used in the subgroup analysis. Higher Hcy levels were detected in the CAVD patients than in the controls in both larger sample size studies (participant number exceeding 200, SMD 0.62, 95% CI 0.35–0.90) and in smaller sample size studies (participant number fewer than 200, SMD 0.45, 95% CI 0.25–0.65) (Figure 4). No between-study heterogeneity was observed in the group of small sample size (I^2^ = 0.0%, *P* = 0.62) (Figure 4). However, heterogeneity remained in the other groups.

Of the 10 studies, two of them divided CAVD patients into two groups: a mild-to-moderate CAVD group and a severe CAVD group. The mild-to-moderate CAVD group exhibited significantly higher levels of plasma Hcy than the controls (SMD 0.13, 95% CI 0.02–0.23) (Supplementary Figure 1). Similarly, the severe CAVD group displayed significantly higher levels of plasma Hcy than the controls (SMD 0.69, 95% CI 0.21–1.17) (Supplementary Figure 2). However, severe CAVD patients did not demonstrate significantly elevated levels of plasma Hcy compared with the mild-to-moderate CAVD patients (SMD 0.83, 95% CI -0.12–1.79) (Supplementary Figure 3).

### Sensitivity analysis and publication bias

**Figure 5:**
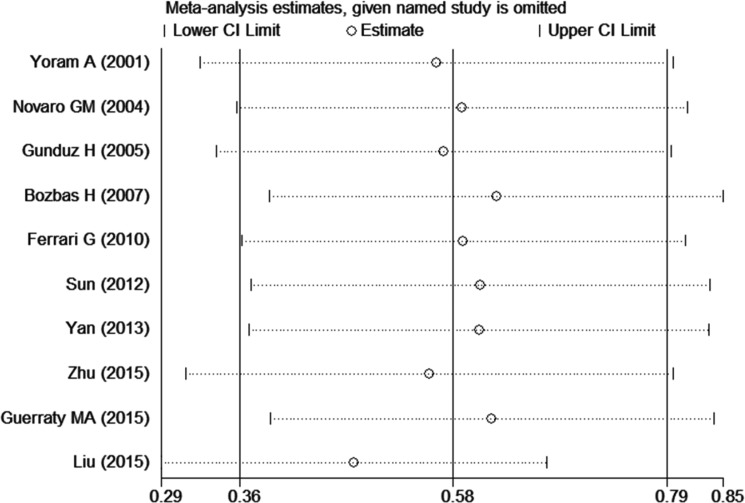
Sensitivity analysis plot of the differences in Hcy levels between CAVD patients and controls Random-effects models were used. The two ends of the dotted lines represented the 95% CI.

In order to determine the stability of our results, we conducted sensitivity analysis. In the sensitivity analysis, little change was found in the pooled SMDs after sequentially removing single studies from the analysis, as shown in Figure 5. The overall SMDs varied from 0.48 (95% CI: 0.29–0.67) to 0.62 (95% CI: 0.39–0.85), which indicated that our results were not being significantly affected by any single study.

**Figure 6:**
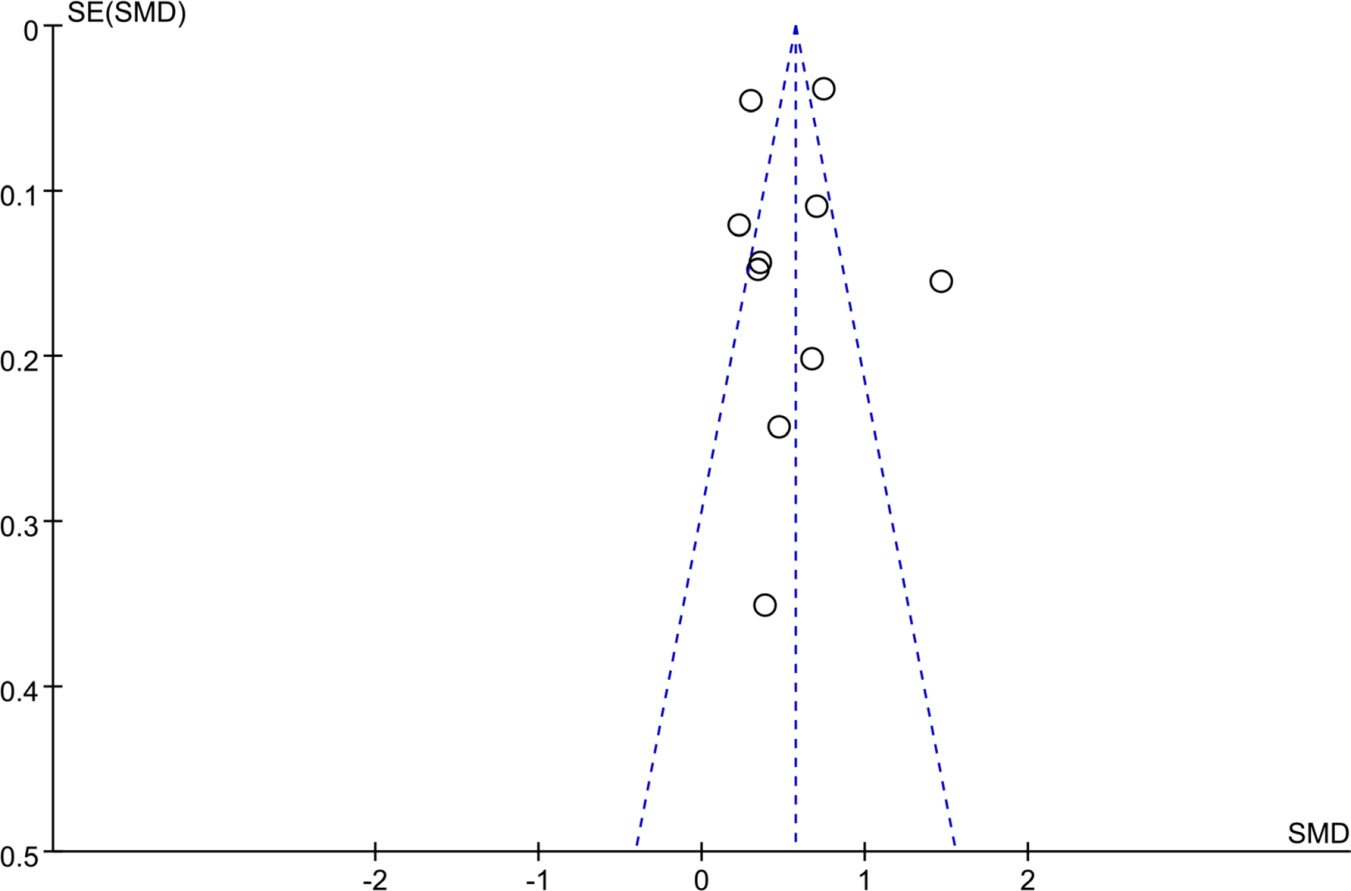
Funnel plot for testing the publication bias of the 10 studies evaluating the association between Hcy levels and CAVD X-axis [standardized mean difference (SMD)] represents effect sizes. Y-axis [SE (SMD)] represents the sample size. Each circle represents an individual study. The dashed line represents 95% CI.

The funnel plot that we created appeared symmetrical (Figure 6), and Begg’s tests (*P* = 1.00) did not reveal evidence of publication bias. This point was further confirmed by Egger’s regression (*P* = 0.99).

### Meta-regression analysis

We performed meta-regression analysis to further investigate the possible sources of heterogeneity. In our univariate meta-regression analysis, the publication year (*P* = 0.68), geographic site of the studies (*P* = 0.33), mean difference in age (*P* = 0.43), percentage of male participants (*P* = 0.28), sample size (*P* = 0.99) and the Newcastle-Ottawa Scale (NOS) score (*P* = 0.47) were not potential sources of heterogeneity, which indicated that none of these parameters exhibited an obvious confounding influence on the association between Hcy levels and CAVD.

## DISCUSSION

Calcific aortic valve disease is considered to be an atherosclerosis-like process that involves multiple factors [21]. However, no drug strategies currently exist to prevent or reduce the progression of CAVD in a clinically significant way [22]. A deep understanding of the risk factors of CAVD provides the foundation for preventive methods and medical therapies for this disease. Therefore, it has become significant to recognize novel biomarkers of CAVD. Previous studies have found that increased Hcy levels were associated with arteriosclerotic outcomes and stroke incidence in elderly people [23] and were capable of increasing the risk of cardiovascular disease [5]. Although elevated Hcy levels have been confirmed to be associated with vascular calcification, such as coronary artery calcification [24] and carotid calcification [25], the relation between Hcy levels and valvular calcification still remains unclear.

To the best of our knowledge, this meta-analysis, which includes 6349 participants spread across 10 case-control studies, is the first study to assess the association between Hcy levels and CAVD. Our results confirmed significantly elevated plasma levels of Hcy in patients with CAVD compared with controls. The elevation was obvious in American and Asian populations. Plasma Hcy levels tended to be higher level in Turkish CAVD patients than in the controls, but the difference was not significant, probably due to the relatively limited number of studies and their small sample sizes. Furthermore, Hcy levels were significantly elevated in patients with both mild-to-moderate CAVD and severe CAVD.

The mechanisms of hyperhomocysteinemia underlying CAVD are not completely understood. There are several plausible explanations for the relation between Hcy levels and CAVD. The pathogenesis of the early-stage CAVD is similar to atherosclerosis [26], which begins with some forms of endothelial dysfunction. Homocysteine can cause endothelial injury [9], thereby damaging the function of the endothelium, including counteracting the adverse effects of blood flow. Under the influence of turbulent flows and oscillatory shear stress, a subendothelial chain reaction is initiated. Furthermore, *in vitro* studies have revealed that elevated Hcy levels prompted the formation of various reactive oxygen free radicals and intensified the oxidative stress-induced injury [27], which is believed to play a significant role in the pathogenesis of CAVD [26]. Additionally, Hcy can promote oxidation of low-density lipoprotein [28] and thereby enhance signaling pathways related to valve calcification [29]. Furthermore, elevated Hcy levels are capable of activating pro-inflammatory factors [30]. It is acknowledged that aortic valve calcification is actively regulated by an inflammatory process, and some pro-inflammatory cytokines can stimulate subsequent mineralization [31].

Plasma Hcy levels can be increased by deficiencies in folic acid, vitamin B_6_, or vitamin B_12_ [32]. To further explore the aberrant Hcy metabolism in CAVD patients, we tried carrying out meta-analyses to investigate the association between these factors and CAVD. However, we could not find published studies regarding vitamin B_6_, vitamin B_12_, folic acid levels, genetic polymorphism of Hcy metabolism enzyme or Hcy-lowering therapy in CAVD patients and controls to carry out a more comprehensive analysis. Additionally, few relevant metabolomics studies were available to potentially shed light on the biological functions of Hcy and systemic perturbations in CAVD patients. A recent meta-analysis indicated a 10% lower risk of stroke and a 4% reduced risk of overall cardiovascular disease with folic acid supplementation [33]. A greater benefit from Hcy-lowering therapy was observed among individuals without preexisting cardiovascular disease and in studies with more significant decreases in Hcy levels [33]. To the best of our knowledge, CAVD, as an independent predictive factor for stroke and myocardial infarction [34], is associated with cardiovascular morbidity and mortality [35]. It remains to be verified whether folic acid supplementation can reduce the risk of cardiovascular events in patients with CAVD. Additional randomized controlled trials are also necessary to examine the effect of Hcy-lowering therapy on the prevention and treatment of CAVD.

Our meta-analysis has several potential limitations. First, significant heterogeneity among the included studies is a chief issue. We used subgroup and meta-regression analyses to explore several potential sources of heterogeneity including geographic site, sample size, publication year, mean difference in age, difference in gender ratio and quality score. However, none of these factors was found to be an important contributor to the heterogeneity. In these studies, the Hcy concentrations were assessed using different methods—including microparticle enzyme immunoassay, fluorescence polarizing enzyme immunoassay and enzyme linked immunosorbent assay (ELISA)—which may explain at least part of the heterogeneity. We also speculated that the sources of heterogeneity could be the stage of CAVD and unknown confounding variables in each study. In addition, the number of studies that reported Hcy levels and different stages of CAVD was limited, and in other eligible studies different stages of CAVD were not defined. Our meta-analyses should accordingly be evaluated with caution. Second, due to data unavailability, we analyzed only a few potential confounding factors in our meta-regression analyses. Other confounding factors such as lipid profile, renal function [36] and diet or medication intake, which may cause bias in the results, were not analyzed. In addition, the included studies did not report clinical measurements in sufficient detail to conduct additional stratified analyses according to different risk factors. Furthermore, CAVD is a predictor of some cardiovascular diseases, such as coronary artery disease [37], which may be associated with increased Hcy levels [38]. This might lead to an overestimation of our results. Additional studies that include more detailed measurements and appropriate controls are necessary to clarify these issues. Third, only studies written in English or Chinese were analyzed, which means that we might have missed other relevant studies. Last but not least, this meta-analysis demonstrated an association but did not demonstrate causation between Hcy levels and CAVD because the included studies were all case-control in design. Future prospective studies are required to demonstrate whether high Hcy levels contribute to aortic valve calcification or that CAVD induces Hcy expression.

Despite these shortcomings, our results shed some light on the association of Hcy levels and CAVD. A relatively large sample size in the present analysis strengthened its statistical power. The stability of our findings was further confirmed by sensitivity analysis.

In conclusion, this study demonstrated higher Hcy levels in CAVD patients compared with healthy controls, indicating that elevated Hcy levels are correlated with CAVD. Nevertheless, additional studies including larger study cohorts and better study designs are necessary to determine the causal role of Hcy in the development of CAVD.

## MATERIALS AND METHODS

### PRISMA guidelines

This systematic review and meta-analysis was complied using the checklist and guidelines from the PRISMA (Preferred Reporting Items for Systematic Reviews and Meta-Analyses) statement [39] listed in Supplementary Table 1.

### Search strategy

A systematic literature search was carried out for original studies published prior to the end of March 2017 evaluating the association of Hcy levels and CAVD in the PubMed, Embase, Web of Science, Cochrane Central Register of Controlled Trials and the Chinese Biomedical Literature databases. Articles published in English or Chinese were included. Potentially relevant articles were identified by various combinations of search terms including the following words: “calcific aortic valve disease,” “aortic valve calcification,” “aortic valve stenosis,” “aortic valve sclerosis,” “homocysteine,” and “hyperhomocysteinemia.” In addition, a manual search of the reference lists of the retrieved articles was conducted to obtain additional eligible studies.

### Selection criteria

The studies were included in this observational meta-analysis if they satisfied the following criteria: 1) an original study with a case-control design examining the relation between Hcy levels and CAVD; 2) CAVD was defined as focal areas of valve leaflet thickening demonstrated by echocardiographic or radiological evidence; 3) the Hcy levels of the cases and controls were explicitly presented as continuous so that the mean and standard deviation (SD) could be estimated; 4) a standardized technique was used to measure Hcy levels at the baseline. Letters, reviews, case-reports and animal studies were excluded. If two or more studies shared the same sample, the study with the complete data was included.

### Data extraction

Baseline data and results from all identified studies were extracted carefully into a spreadsheet. The following items were included: the first author’s name, the year of publication, the country in which the study was conducted, the sample size, the age range, the gender and Hcy levels in the case and control groups. Study selection and data extraction were carried out carefully by two investigators independently, and disagreements were resolved via discussion.

### Quality assessment

The quality of studies accepted for the meta-analysis was evaluated using the Newcastle-Ottawa Scale (NOS) [40]. Selection, comparability and exposure were considered in our quality assessment. The maximum score was 9 points. Studies with a score of at least 7 were considered to be of high quality, and studies with a score ranging from 4–6 were defined as being of medium quality.

### Statistical methods

Because various methods for measuring Hcy levels were used in the included studies, we used the SMD but not the weighted mean difference (WMD), with the 95% CI in this analysis. We evaluated heterogeneity using the Q and I^2^ statistics. I^2^ provides an estimate of the amount of variance attributable to between-study heterogeneity rather than chance. The difference was considered to be significant if P_Q_< 0.1 or I^2^ > 50%, and a random effect model was conducted. Otherwise, a fixed effect model was used. The corresponding subgroup analyses were performed by predefined criteria based on the geographic site (the United States of America, Asia or Turkey) and the sample size (≥ 200 or < 200). If significant heterogeneity was observed in the meta-analysis, univariate meta-regression analysis was conducted to explore the possible sources of heterogeneity. The publication year, geographic site, mean difference in age, difference in gender ratio, sample size and quality score were used as covariates. Sensitivity analysis was performed to assess the stability of our results by sequentially omitting studies. Furthermore, we estimated potential publication bias using funnel plots, Begg’s test and Egger’s regression. Review Manager (version 5.3; Copenhagen: The Nordic Cochrane Centre, The Cochrane Collaboration, 2014) and STATA 12.0 (Stata Corporation, College Station, TX, USA) were used.

## SUPPLEMENTARY MATERIALS FIGURES AND TABLE





## Abbreviations

Hcy: homocysteine
CAVD: calcific aortic valve disease
CBM: the Chinese Biomedical Literature databases
SMD: standardized mean difference
95% CI: 95% confidence interval
AVSc: aortic valve sclerosis
AVS: aortic valve stenosis
PRISMA: Preferred Reporting Items for Systematic Reviews and Meta-Analyses
SD: standard deviation
NOS: Newcastle-Ottawa Quality Assessment Scale
