# Supplementary material for: Association between homocysteine levels and calcific aortic valve disease: a systematic review and meta-analysis
Source: Oncotarget. 2018 Jan 3;9(9):8665–74. doi: 10.18632/oncotarget.23938 (PMC5823577; doi:10.18632/oncotarget.23938)
Supplement: Supplementary file 1 [file oncotarget-09-8665-s001.pdf]

Association between homocysteine levels and calcific aortic valve disease: a systematic review and meta-analysis

SUPPLEMENTARY MATERIALS

	Mild-to-moderate CAVD			Controls				Std. Mean Difference
Study or Subgroup	Mean	SD	Total	Mean	SD	Total	Weight	IV, Fixed, 95% CI
Novaro GM 2004	11.13	2.71	32	10.67	4.98	27	4.1%	0.12 [-0.40, 0.63]
Guerraty MA 2015	14.17	4.86	515	13.54	5.03	1023	95.9%	0.13 [0.02, 0.23]
**Total (95% CI)**			**547**			**1050**	**100.0%**	**0.13 [0.02, 0.23]**

Heterogeneity: Chi² = 0.00, df = 1 (P = 0.97); I² = 0%
Test for overall effect: Z = 2.38 (P = 0.02)

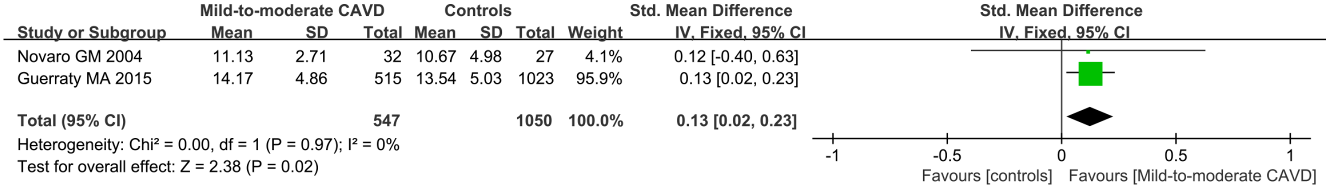

Supplementary Figure 1: Forest plot of the differences in Hcy levels between mild-to-moderate CAVD patients and controls.

	Severe CAVD			Controls				Std. Mean Difference
Study or Subgroup	Mean	SD	Total	Mean	SD	Total	Weight	IV, Random, 95% CI
Novaro GM 2004	15.7	4.12	17	10.67	4.98	27	31.1%	1.06 [0.41, 1.71]
Guerraty MA 2015	16.55	7.06	426	13.54	5.03	1023	68.9%	0.53 [0.41, 0.64]
**Total (95% CI)**			**443**			**1050**	**100.0%**	**0.69 [0.21, 1.17]**

Heterogeneity: Tau² = 0.08; Chi² = 2.48, df = 1 (P = 0.12); I² = 60%
Test for overall effect: Z = 2.82 (P = 0.005)

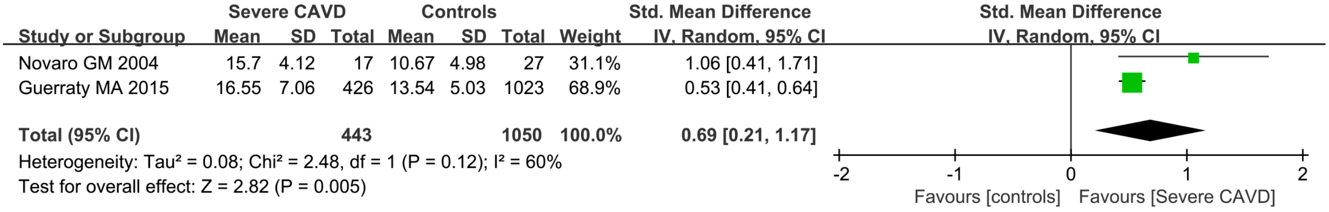

Supplementary Figure 2: Forest plot of the differences in Hcy levels between severe CAVD patients and controls.

	Severe CAVD			Mild-to-moderate CAVD				Std. Mean Difference
Study or Subgroup	Mean	SD	Total	Mean	SD	Total	Weight	IV, Random, 95% CI
Guerraty MA 2015	15.7	4.12	17	11.13	2.71	32	44.4%	1.38 [0.73, 2.03]
Novaro GM 2004	16.55	7.06	426	14.17	4.86	515	55.6%	0.40 [0.27, 0.53]
**Total (95% CI)**			**443**			**547**	**100.0%**	**0.83 [-0.12, 1.79]**

Heterogeneity: Tau² = 0.42; Chi² = 8.32, df = 1 (P = 0.004); I² = 88%
Test for overall effect: Z = 1.71 (P = 0.09)

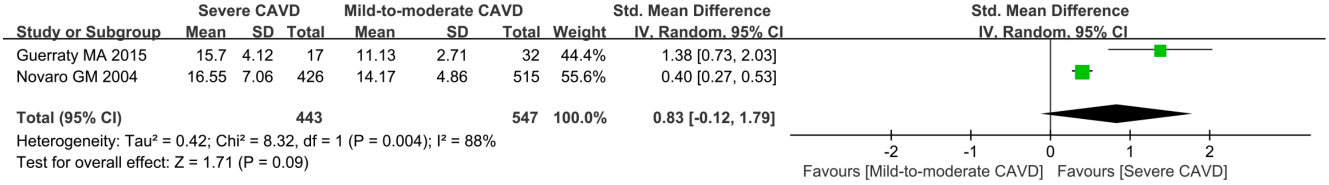

Supplementary Figure 3: Forest plot of the differences in Hcy levels between severe CAVD patients and mild-to-moderate CAVD patients

Supplementary Table 1: Checklist from the PRISMA. See Supplementary_Table_1
